# Impacts of air pollution and meteorological conditions on dry eye disease among residents in a northeastern Chinese metropolis: a six-year crossover study in a cold region

**DOI:** 10.1038/s41377-023-01207-1

**Published:** 2023-07-27

**Authors:** Cheng-Wei Lu, Jing Fu, Xiu-Fen Liu, Zhi-Hua Cui, Wei-Wei Chen, Li Guo, Xiao-Lan Li, Yu Ren, Fei Shao, Li-Na Chen, Ji-Long Hao

**Affiliations:** 1 Opthalmology Department, The First Hospital of Jilin University, Changchun, 130021 China; 2 Key Laboratory of Wetland Ecology and Environment, Northeast Institute of Geography and Agroecology, Chinese Academy of Sciences, Changchun, 130102 China; 3 College of New Energy and Environment, Jilin University, Changchun, 130021 China; 4 China College of Biological and Agricultural Engineering, Jilin University, Changchun, 130022 China; 5 Shenyang Institute of Atmospheric Environment, China Meteorological Administration, Shenyang, 110166 China

**Keywords:** Lasers, LEDs and light sources, Other photonics

## Abstract

The purpose of this study is to explore the associations among dry eye disease (DED), air pollution, and meteorological conditions in the cold region of a northeastern Chinese metropolis (i.e., Changchun). Data on ambient air pollutants and meteorological parameters as well as diagnosed DED outpatients during 2015–2021 were collected. The associations between DED and environmental factors were analysed at multiple time scales using various statistical methods (i.e., correlation, regression and machine learning). Among the 10,809 DED patients (21,617 eyes) studied, 64.60% were female and 35.40% were male. A higher frequency of DED was observed in March and April, followed by January, August and October. Individual and multiple factor models showed the positive importance of particles with aerodynamic diameters <10 μm (PM_10_), carbon monoxide (CO), and ozone (O_3_) among normal air pollutants and air pressure (AP), air temperature (AT) and wind speed (WS) among normal meteorological parameters. Air pollutants (PM_10_, nitrogen dioxide: NO_2_) and meteorological parameters (AT, AP) have combined impacts on DED occurrence. For the first time, we further explored the associations of detailed components of atmospheric particles and DED, suggesting potential emission sources, including spring dust from bare soil and roads and precursor pollutants of summer O_3_ formation from vehicles and industry in Northeast China. Our results revealed the quantitative associations among air pollutants, meteorological conditions and DED outpatients in cold regions, highlighting the importance of coordinated policies in air pollution control and climate change mitigation.

## Abstract (web summary)

Impacts of air pollution and meteorological conditions on dry eye disease among residents in a northeastern Chinese metropolis: A six-year crossover study in a cold region.

## Graphical abstract

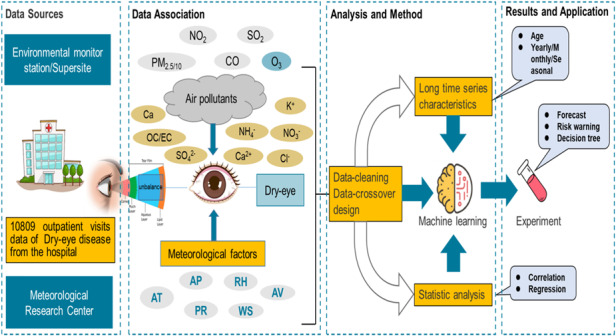

## Introduction

Dry eye disease (DED) is a multifactorial chronic disorder of the tears and ocular surface that has been a growing public health concern around the world in recent years. DED was diagnosed when a patient had DED symptoms and at least one objective DED sign (tear film abnormalities [tear film break-up time ≤ 7 s] or positive ocular surface staining [Oxford grade ≥ 1]^)1^. It is characterized by visual impairment, a burning sensation, pain, itching, and tear film instability with latent impairment to the ocular surface^2^. According to the China Dry Eye Expert Consensus (2020) statistics, the incidence of DED in Asia ranks highest in the world, among which the incidence in China is ~21–30%^3^. Based on this calculation, the number of DED patients in China is ~400 million, with an average of one to two patients for every five people. Worldwide, the prevalence of DED reported by numerous studies conducted in several countries and regions was relatively different, ranging from 5 to 50% by reason of disease definition, population characteristics, and other risk factors^4–11^. Almost 16.4 million adults (6.8%) have been diagnosed with DED in the United States, and the financial burden of DED management has been estimated to be nearly $55 billion per year^12^.

DED is influenced by environmental factors, such as wind, low humidity, high altitude, high temperature, and air pollution^13–15^. An increasing number of people are suffering from the increased adverse effects of air pollution owing to the cost of industrialization, seen currently in the rapid development of industrialization and urbanization in China. Air pollution is becoming a main source of morbidity for human beings^16^. Frequent exposure of the ocular surface to air pollutants increases the vulnerability of the eyes to these pollutants^17^. China has suffered severe air pollution as well as climate change in recent decades^16^. A few studies have evaluated the effects of air pollution on DED globally^1,2,18,19^. Evaluations of air pollution levels and their effects on DED have been conducted in Hangzhou^2^. Among the 5062 DED patients studied, significant associations were detected between outpatient visits for DED and an increase of 10 μg m^−3^ in the concentrations of fine particulate matter with a median aerometric diameter of <2.5 μm (PM_2.5_), fine particulate matter with a median aerometric diameter of <10 μm (PM_10_), nitrogen dioxide (NO_2_), carbon monoxide (CO), and sulfur dioxide (SO_2_). Retrospective data from South Korea indicate that lower humidity and higher ozone (O_3_) levels are associated with an increased prevalence of DED^18^. Pathologic effects on the stability of tear film have also been observed in a low humidity-controlled chamber^20^. Relative humidity could correlate with signs and symptoms of DED. A report from the Dry Eye Assessment and Management Study showed that DED signs differed according to local humidity and climate levels. However, airborne pollutants were not associated with detrimental DED features with the exception of NO_2_^21^. Even though a few studies have been conducted to that effect, there are still many countries and regions that remain unsurveyed, and the potential associations among DED, air pollution, and meteorological conditions in the cold region of China with the most prominent climate change and typical pollution characteristics are far from understood. In addition, emission sources and management of air pollutants are critical for the prediction and prevention of DED and for the development of health-based environmental quality standards, which have not been reported to date.

As a cold region with high latitude, Northeast China suffers typical air pollution, with the number of haze days increasing sharply during the past 50 years^22^. The centrally distributed heating supply in winter and agricultural open burning have also added to this complication. Furthermore, the annual mean air temperature (AT) rise rate (0.31 °C per 10 years) was significantly higher than that in other regions of China^23^. All these factors lead to diversified air pollution and climate change contributors in Northeast China^24^. However, to date, no study has been conducted on the relative impact of air pollutants and meteorological variables on DED cases in cities or metropolises in high-latitude cold regions in Northeast China. In this study, the incidence of DED, air pollutant data, and meteorological data in a northeastern Chinese metropolis (i.e., Changchun) in the last six years were collected and analysed to determine whether the prevalence of DED is associated with meteorological conditions or air pollution. To our knowledge, this is the first study to illuminate the underlying impacts of air pollutants and climate factors on DED in the cold region of Northeast China. We believe it can be of aid in a wiser allocation of stretched resources for DED control and prevention.

## Results

### Temporal variation in DED and environmental factors

**Fig. 1:**
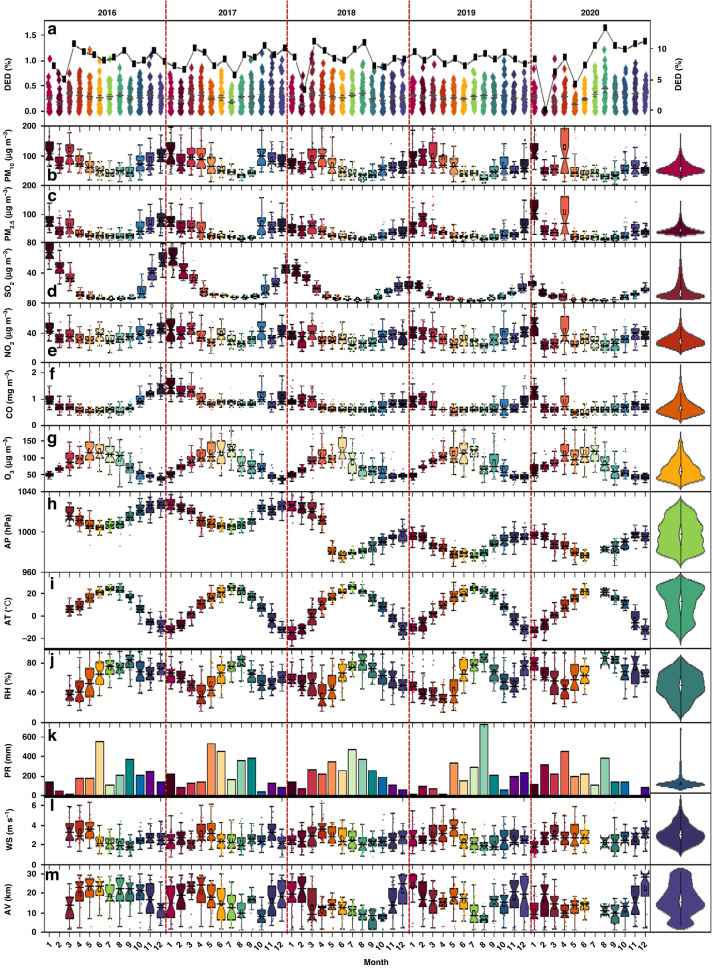
Monthly variations in dry eye disease (DED) and ambient environmental factors (i.e., meteorological factors and air pollutants) from 2016 to 2020. DED data on outpatient visits from July 15, 2015, through July 30, 2021, are only displayed for the whole year (2016–2020). Proportion of outpatient visits for DED per day of the whole month (**a**, main coordinate, %) and the proportion of outpatient visits in the current month of the whole year (**a**, secondary coordinate, %). Monthly data of these variables were arithmetic mean values of daily data (**b**–**m**) for air pressure (AP, hPa), air temperature (AT, °C), relative humidity (RH, %), wind speed (WS, m s^−1^), air visibility (AV, km), PM_2.5_ (μg m^−3^), PM_10_ (μg m^−3^), O_3_ (μg m^−3^), NO_2_ (μg m^−3^), SO_2_ (μg m^−3^) and CO (mg m^−3^), while precipitation (PR, mm) is the cumulative value for each month. The violin plot on the right represents the distribution of monthly data and its probability density for five consecutive years (2016–2020). The box plot shows the data averages, medians, fluctuations, upper and lower quartiles, and outliers

Monthly dynamics, statistical data of DED and environmental factors (i.e., air pollutants and meteorological factors) throughout the study period (2016–2020) are shown in Table 1 and Fig. 1. The monthly prevalence rate of DED is expressed by the normalized index, which refers to the percentage of the number of outpatient visits in the current month and that in the entire year. Except in February 2020, when the hospital was locked down due to COVID-19 in the city of Changchun, the monthly prevalence rate of patients with DED ranged from 3.24 to 13.25%, showing a higher frequency in March and April, followed by January, August and October, and a lower frequency in other months. Among the environmental factors, the monthly trends of AT, wind speed (WS) and visibility (AV) over the years were similar and relatively stable. The annual mean concentrations of PM_2.5_, PM_10_, SO_2_, NO_2_, and O_3_ were 40.6, 68.7, 17.3, 34.3, and 79.4 μg m^−3^, respectively, and CO was 0.8 mg m^−3^. Monthly concentrations of pollutants (PM_2.5_, PM_10_, SO_2_, NO_2_) were higher in January, March and April, followed by October and November. Since 2018, sharp decreases in particulate matter (PM_2.5_, PM_10_) have been observed, and annual NO_2_, SO_2_ and CO concentrations have shown decreasing trends. Moreover, O_3_ only showed a downwards trend in 2018. The annual mean air pressure (AP), AT, relative humidity (RH), precipitation (PR), WS and AV during the study period were 1000.3 hPa, 7 °C, 61.3%, 208.75 mm, 2.68 m s^−1^, and 18.33 km, respectively. The AP was low when the temperature was high in June and July. The maximum relative humidity occurred in August, when rainfall was at its highest level. Moreover, the WS was strongest in April and May, followed by November.

**Table 1:** Monthly statistics of air pollutants, meteorological factors and DED in the city of Changchun in Northeast China from 2016 to 2020

Month	DED (%)	Air pollutants						Meteorological factors					
		SO_2_ (μg m ^−3^)	PM_2.5_ (μg m ^−3^)	PM_10_ (μg m ^−3^)	NO_2_ (μg m ^−3^)	CO (mg m ^−3^)	O_3_ (μg m ^−3^)	AP (hPa)	AT (°C)	RH (%)	PR (mm)	WS (m s^−1^)	AV (km)
January	9.2 ± 1.9	45 ± 18	80 ± 22	103 ± 17	45 ± 5	1.14 ± 0.2	51 ± 5	1014 ± 15	−13 ± 2	63 ± 9	125 ± 66	2.40 ± 0.3	20.28
February	5.3 ± 3.4	32 ± 14	54 ± 16	76 ± 13	34 ± 5	0.92 ± 0.2	72 ± 8	1010 ± 16	−8 ± 5	53 ± 6	121 ± 95	2.74 ± 0.2	19.64
March	11.4 ± 1.8	22 ± 9	68 ± 20	106 ± 15	41 ± 3	0.94 ± 0.2	95 ± 8	1006 ± 16	3 ± 3	46 ± 6	136 ± 94	2.93 ± 0.4	17.57
April	9.7 ± 1.5	10 ± 4	35 ± 8	87 ± 17	31 ± 4	0.68 ± 0.2	104 ± 3	999 ± 15	11 ± 3	43 ± 9	199 ± 145	3.32 ± 0.2	19.82
May	7.4 ± 0.7	7 ± 2	24 ± 3	67 ± 18	29 ± 2	0.61 ± 0.1	113 ± 7	990 ± 14	18 ± 2	52 ± 6	317 ± 128	3.34 ± 0.3	18.01
June	7.6 ± 1.1	6 ± 2	22 ± 3	46 ± 4	34 ± 4	0.66 ± 0.1	117 ± 3	989 ± 14	22 ± 1	66 ± 2	326 ± 151	2.46 ± 0.1	17.06
July	7.8 ± 1.7	5 ± 2	20 ± 4	41 ± 5	27 ± 3	0.65 ± 0.1	104 ± 16	990 ± 13	24 ± 2	77 ± 4	226 ± 137	2.36 ± 0.1	18.75
August	10.4 ± 2.6	5 ± 2	16 ± 4	35 ± 7	26 ± 3	0.66 ± 0.1	75 ± 12	991 ± 13	21 ± 2	81 ± 4	407 ± 172	2.20 ± 0.2	13.46
September	7.1 ± 0.8	7 ± 2	21 ± 5	47 ± 6	32 ± 2	0.70 ± 0.1	70 ± 8	997 ± 13	16 ± 3	69 ± 6	270 ± 93	2.33 ± 0.3	16.24
October	9.2 ± 2.7	13 ± 4	43 ± 14	72 ± 18	39 ± 3	0.88 ± 0.2	59 ± 9	1004 ± 15	5 ± 4	62 ± 8	125 ± 68	2.54 ± 0.0	16.33
November	8.3 ± 0.9	23 ± 10	46 ± 16	68 ± 15	36 ± 3	0.85 ± 0.2	45 ± 2	1006 ± 14	-5 ± 4	59 ± 6	134 ± 81	2.93 ± 0.3	20.43
December	6.5 ± 1.6	32 ± 16	59 ± 16	76 ± 18	38 ± 9	1.01 ± 0.3	48 ± 14	1008 ± 15	−10 ± 2	65 ± 8	119 ± 64	2.56 ± 0.2	22.45

*DED* dry eye disease, *AP* air pressure, *AT* air temperature, *RH* relative humidity, *AV* air visibility, *PR* precipitation, *WS* wind speed

### Determination of dominant environmental factors for DED

We first analysed the Pearson’s correlation coefficients between morbidity data and air pollutants as well as meteorological factors based on the monthly mean values from 2016 to 2020. Significant positive associations were observed between PM_10_ and DED. O_3_, CO, NO_2_ and PM_2.5_ were positively correlated with DED. The meteorological factors of AT and WS were also positively correlated with DED. In contrast, there was a negative correlation between DED and RH, PR, and AV. As expected, not all factors were simply significantly correlated with each other, with further analysis of the grey correlation shown in Table 2. The monthly DED rate showed grey relational grades (largest to smallest) as follows: AP (0.89) > NO_2_ (0.89) > WS (0.88) > CO (0.86) > PM_10_ (0.86) > RH (0.85) > O_3_ (0.84) > AV (0.83) > PM_2.5_ (0.80) > PR (0.77) > SO_2_ (0.73) > AT (0.53). Except for AT, strong grey correlations were found among the other factors in November of this study. Similarly, strong correlations were determined between pollutant indicators and air temperature as well as precipitation in March and April. In addition, AP, RH and WS were strongly correlated with DED in June and July. In May, there were high correlations with PM_10_ (*r* = 0.91), NO_2_ (*r* = 0.96), CO (*r* = 0.90), AV (*r* = 0.92), AP (*r* = 0.94), and RH (*r* = 0.93).

**Table 2:** Grey correlation analysis between air pollutants and meteorological factors of mean DED by month from 2016 to 2020

Indices		Air pollutants						Meteorological factors					
		SO_2_	PM_2.5_	PM_10_	NO_2_	CO	O_3_	AP	AT	RH	PR	WS	AV
Grey relational grade (ranking)		0.73(11)	0.80(9)	0.86(5)	0.89(2)	0.86(4)	0.84(7)	0.89(1)	0.53(12)	0.85(6)	0.77(10)	0.88(3)	0.83(8)
Grey relational coefficient	January	0.61	0.70	0.81	0.86	0.83	0.79	0.89	0.36	0.89	0.79	0.88	0.87
	February	0.60	0.70	0.80	0.83	0.78	0.84	0.80	0.47	0.79	0.80	0.80	0.76
	March	0.80	0.89	0.92	0.84	0.80	0.89	0.83	0.61	0.75	0.73	0.88	0.81
	April	0.76	0.83	0.84	0.91	0.86	0.89	0.89	0.85	0.77	0.80	0.90	0.85
	May	0.78	0.86	0.91	0.96	0.90	0.77	0.94	0.53	0.93	0.66	0.82	0.92
	June	0.76	0.82	0.87	0.91	0.88	0.75	0.92	0.44	0.90	0.79	0.95	0.87
	July	0.73	0.81	0.84	0.89	0.85	0.81	0.90	0.39	0.86	0.80	0.91	0.76
	August	0.65	0.67	0.70	0.79	0.81	0.84	0.88	0.48	0.87	0.69	0.80	0.71
	September	0.77	0.81	0.88	0.95	0.96	0.98	0.93	0.52	0.84	0.74	0.92	0.90
	October	0.81	0.91	0.95	0.92	0.91	0.86	0.89	0.81	0.87	0.79	0.88	0.86
	November	0.83	0.88	0.94	0.94	0.94	0.80	0.96	0.54	0.94	0.88	0.95	0.92
	December	0.67	0.74	0.82	0.81	0.81	0.87	0.87	0.42	0.83	0.77	0.88	0.79

The number in parentheses for grey relational grade represents the importance ranking between DED and environmental factors

*AP* air pressure, *AT* air temperature, *RH* relative humidity, *AV* air visibility, *PR* precipitation, *WS* wind speed

Multivariate stepwise regression analysis suggested that the potential risk factors for DED were the meteorological factor of AT and air pollutants, including PM_10_ and O_3_. The results of the *F* test (*F* = 7.06) obtained a significant level of 1% (*p* < 0.0001) with rejection of the null hypothesis (regression coefficient = 0). For the collinearity of variables, VIFs were all <10, so the model was well constructed with no multicollinearity problem. The formula of the model is as follows: Y = 5.344 + 0.163*AT + 0.071*PM_10_ − 0.038*O_3_. The pollutant O_3_ was not positively associated with DED, a finding that was contrary to the results of the single correlation. Because of the small amplitude of the variation range of O_3_, as observed during the interyear variation analysis, the adverse effects of O_3_ are not observed for DED in the short term (intrayear).

**Fig. 2:**
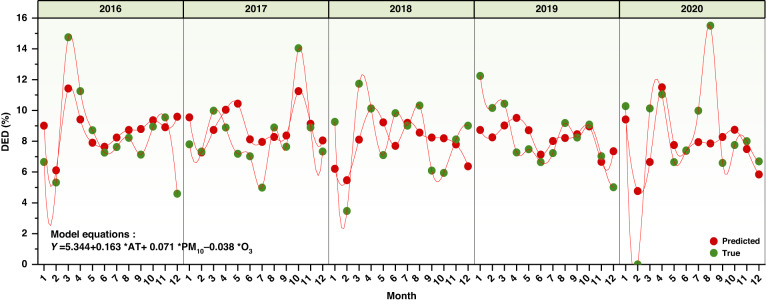
Logistic regression model simulating trends and equations. The green solid circles represent true values, while the red solid circles are predicted values based on the simulated model equation

According to the model fitting, the monthly change trend from 2016 to 2020 was compared with the real data analysis (Fig. 2). The simulated DED data showed a peak value in March and April, reaching a maximum of 11.7%, which may be an important node for potential risk factors. However, the second peak value of DED occurred in September and October, except for the obvious decline in 2018, which may be related to the local Straw Burning Ban Policy in 2018.

**Fig. 3:**
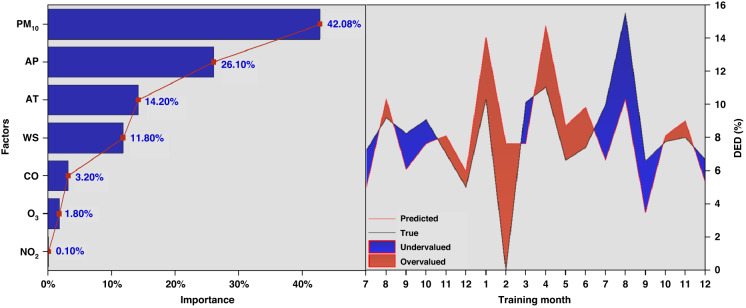
Importance ratio of the main environmental factors (left) and modelling training prediction for DED (right). The training ratio is 0.7, and the training month in the figure on the right represents the remaining 30% of the month’s data simulation forecast

We used the machine learning model to investigate the association between the main factors and outpatient visits for DED (Fig. 3). Based on logistic regression and grey correlation analysis, seven main factors (i.e., AT, PM_10_, O_3_, AP, WS, CO and NO_2_) were screened for training. The regression model for decision number was established by dataset training and obtaining the structure of the decision tree. MSE, RMSE and MAE were used to determine the internal nodes and were divided into branching characteristics. The calculation results for the largest proportion were PM_10_ (42.8%) in each importance feature. Both AT (14.2%) and WS (11.8%) accounted for more than 10% of the importance of DED. The contributions of CO, O_3_ and NO_2_ were relatively low, which may be related to the cross-mutual effect of factors. Furthermore, 70% of the dataset was trained in this study, with 30% for predictions (July–December 2019 and 2020). The prediction trend was consistent with the observed results but significantly higher than the real value in November–December 2019 and February 2020.

### Joint effects of environmental factors on DED

**Fig. 4:**
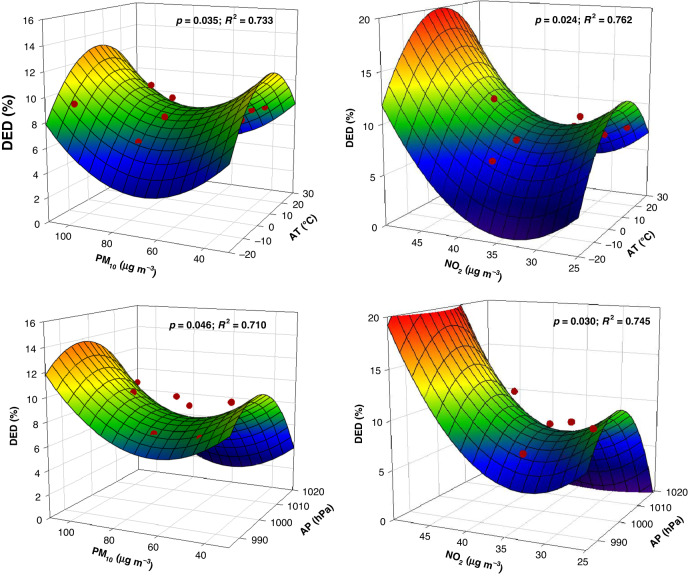
Synergistic effects of individual environmental factors (i.e., air pollutants and meteorological factors) on DED. The red solid circles represent the synergistic environmental factors corresponds to DED, while the colored nets represent the change trend of DED in 3D simulation from hight to low (blue to red)

Multieffect fitting with the major factors in machine learning modelling was analysed. According to the importance of these factors (e.g., primary pollutants and major meteorological indices), four factors (i.e., PM_10_, NO_2_, AT, and AP) were screened by significant fitting (Fig. 4). Consistent with our analysis using single factors, PM_10_ and AT were strongly associated (*p* = 0.035; *R*^2^ = 0.733) with DED after adjusting for certain factors. With an increase in the concentration of PM_10_ and the temperature (15°C–20°C), DED (12.9%) appears to have the greatest influence. Moreover, AP was also one of the co-operators with PM_10_ (*p* = 0.046; *R*^2^ = 0.710) to affect DED (13.8%), and its influence was higher than that of AT. However, NO_2_ played a small role in the importance of machine learning modelling but significantly fit in the coeffecting of major climate factors (i.e., AT and AP) on DED. Our results suggested that the combined effects of these factors (i.e., NO_2_ and AT; NO_2_ and AP) affect the occurrence of DED by more than 70%.

### The link between the chemical components of PM_2.5_ and DED

**Fig. 5:**
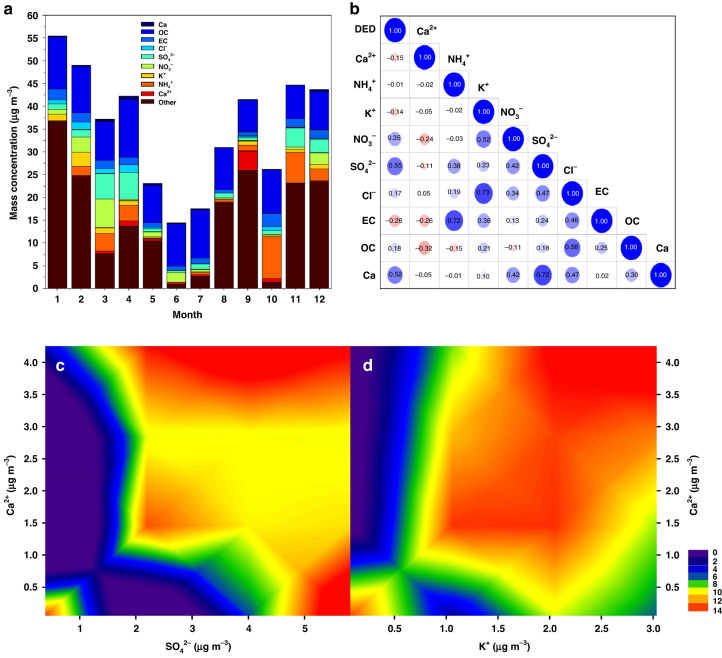
The relationships between the chemical components of PM_2.5_ and DED. Monthly variations in atmospheric PM_2.5_ concentrations and their chemical components at the super monitoring station in the city of Changchun (**a**), correlations among these components (**b**), and joint relationship between major components and DED (**c**–**d**). Monthly figures are calculated using hourly scale data

The real-time chemical components of ambient PM_2.5_ observed in 2020-2021 were used to determine the possible link to DED. The differences in the total PM_2.5_ concentrations and their major components were distinct in each month (i.e., the average of each month from 2016 to 2020) (Fig. 5a). The PM_2.5_ concentrations were lower during May–July and higher during January and February. Considering these components, significantly higher SO_4_^2−^ (5.73 μg m^−3^) and Ca (0.61 μg m^−3^) concentrations were detected in March and April, as well as NO^3−^ (6.28 μg m^−3^) in March and organic carbon (OC) (12.77) and Cl^-^ (1.72) in April. In addition, the ratios of Ca^2+^ (4.25) and NH_4_^+^ (9.27) in the PM_2.5_ samples significantly increased in September and October, respectively. We also analysed the correlations between DED and the main chemical components of PM_2.5_ in Changchun (Fig. 5b). Positive correlations were observed between DED and Ca (*p* = 0.008, *r* = 0.52) and SO_4_^2−^ (*p* = 0.006, *r* = 0.55). In contrast, there was a significant (*p* < 0.05) negative correlation between DED and EC (*p* = 0.03, *r* = −0.26) and K^+^ (*p* = 0.04, *r* = −0.14). Then, the primary components were screened for multifactor analysis (Fig. 5c, d). Increased levels of Ca^2+^ and SO_4_^2−^ significantly worsened DED. Moreover, with increasing Ca^2+^ and K^+^ concentrations, the incidence of DED appeared to increase significantly.

## Discussion

The eye, one of the few human organs constantly exposed to the external environment, is directly affected by air pollution and climate change. DED is a common ocular surface disease that causes eye discomfort, changes in visual acuity, and even blindness^1,2^. In recent years, particulate matter (PM)-induced DED has become a dramatic health concern worldwide^1,2^. According to the World Health Organization, PM, O_3_, NO_2_, and SO_2_ are the most significant pollutants^25^. Our study, for the first time, evaluated the links among air pollutants (PM_10_, PM_2.5_, CO, O_3_, NO_2_, and SO_2_), meteorological parameters (AP, AT, WS, PR, RH, and AV) and the clinical characteristics of DED in cold regions of China. Our results showed that several air pollutants (PM_10_, O_3_, CO) and AT are positively correlated with DED, indicating that these factors may promote the onset of or aggravate DED. However, the adverse effects of air pollutants are complicated, especially when multiple factors work together. Furthermore, we established the joint effect of important environmental factors, including air pollutants and meteorological factors, on DED and analysed the characteristics and potential emission sources of cold regions, which can be of great significance for public care and local regional atmospheric environment management and planning.

Mounting evidence has shown that PM exposure is correlated with clinical changes to the ocular surface. PM_2.5_ increased the number of mast cells, inflammatory cytokine expression, such as Interleukin-1β (IL-1β), Interleukin-6 (IL-6), tumour necrosis factor-α (TNF-α), nuclear factor kappa-B (NF-κB) and NF-κB p65, and apoptosis and decreased cell viability, migration, and the number of corneal desmosomes/microvilli in the cornea, leading to abnormal cell proliferation and differentiation and disrupted cellular membrane integrity^26,27^. PM_2.5_ was associated with DED in the Korean population, while PM_10_ and NO_2_ did not contribute to DED. In contrast to previous studies, we highlighted the importance of atmospheric PM_10_ (i.e., the sum of PM_2.5_ and coarse particulates with diameter sizes from 2.5 to 10 microns) on DED, which may weaken the effect of PM_2.5_ for several reasons. First, the climate in the cold regions of Northeast China is drier, which is more likely to cause DED than humid regions and also induces more coarse particulate emissions in PM_10_^28^. Second, road dust and building dust are some of the main sources of urban PM_10_. Compared with cities in southern China, the level of fine air pollution management and measures in northern cities are relatively weak, increasing the daily PM_10_ emissions^29^. Third, our previous studies have shown that as the main production area of dry crops, PM_10_ emissions during windblown dust and soil tilling periods are much higher in Northeast China than in southern regions dominated by paddy fields^30^. In addition, due to its proximity to deserts in the Inner Mongolia Autonomous Region and Mongolia, the frequency of sandstorms in the studied cold region is much higher than that in other regions, which inevitably results in higher PM_10_ input than PM_2.5_.

One of the hallmarks of DED is a decrease in the number of conjunctival goblet cells (CGC), which can be depleted upon ocular surface inflammation or damage. It has been reported that PM_10_ reduced the number and induced hypoplasia of goblet cells in the conjunctiva^31^, leading to abnormal cell proliferation and differentiation of the ocular surface. Previous studies proved that PM_10_ exposure may disrupt epithelial integrity and cause dramatic damage to the corneal/conjunctiva epithelium, as shown by corneal fluorescein and rose Bengal/Lissa mine green staining^27^. The above results indicate that PM_10_ may disrupt epithelial integrity and break down the ocular surface barrier, resulting in DED. In our study, PM_10_ was also found to be positively correlated with DED, which was consistent with previous studies.

One of the strengths of this study is that it is the first to explore the potential emission sources of atmospheric pollutants affecting DED. Our previous studies have shown that major emission sources of ambient PM_2.5_ include secondary aerosols (39.1%), biomass burning (20.0%), supply heating (17.9%), road/soil dust (13.6%) and traffic (9.3%) in the city of Changchun^32^. The biomass burning portion was characterized by an obviously high concentration of K^+^. Road and soil dust provided the primary mineral species of Ca and ion Ca^2+^. In this study, we found that elevated levels of Ca^2+^ and K^+^ promoted the prevalence of DED, indicating potential sources from agricultural burning and dust emissions. Furthermore, the close link between DED and high concentrations of SO_4_^2−^ indicates the role of coal burning and industrial emissions. Therefore, these findings suggest that three typical emission sources (i.e., agricultural burning, coal burning and dust) in cold regions could increase the incidence of DED.

Gaseous pollutants (e.g., CO, O_3_, NO_2_ and SO_2_) may also play an important role in the prevalence of DED. In our study, DED was positively correlated with O_3_, CO and NO_2_, but the importance of SO_2_ was relatively lower than that of other gaseous pollutants. O_3_, as a powerful oxidant, has been reported to be correlated with adverse health effects. Higher O_3_ exposure was found to be associated with DED symptoms, increased Ocular Surface Disease Index (OSDI) scores and decreased tear secretion in DED patients^1^. It was further reported that O_3_ upregulated the expression of IL-1β, IL-6, and Interleukin-17 (IL-17) in tears and downregulated conjunctival goblet cell density in mouse models, leading to ocular surface discomfort and inflammation^33^. NO_2_ exposure was positively correlated with DED outpatient visits^19^ and increased risk of DED^34^. Higher NO_2_ exposure was negatively correlated with tear secretion and TBUT and positively correlated with increased IL-6 and Interleukin-8 (IL-8) expression in tears and aggravated MG dysfunction^18,19,21,25^. In our study, O_3_ and NO_2_ were found to be positively correlated with DED, which is consistent with a previous report. In the past 10 years, air particle pollution in Northeast China has been decreasing gradually, but the number of O_3_ pollution days has increased significantly, which needs to be given more attention^35^. Unlike particulate matter, O_3_ is a secondary pollutant formed mainly by photochemical reactions of precursors (NO_2_ and volatile organic chemicals) in the atmosphere and is affected by weather conditions, precursor concentration and regional transport. However, there is a two-way feedback relationship between air pollution and climate change. On the one hand, air pollution can affect climate change; on the other hand, the affected climate can change the generation and diffusion of air pollutants^36^. However, the relationship between climate change and air pollution is still being explored, for example, atmospheric-scale climate factors and regional tropospheric O_3_, the spatial difference in climate change on O_3_ precursors and tropospheric O_3_ concentrations, and the impact of climate change on particles. Therefore, the complexity of these factors should be considered in further studies. It is necessary to further carry out multi-regional, multi-component and multi-period synchronous studies.

CO was reported to be inversely correlated with the incidence of DED in a study conducted in South Korea^37^. In contrast, the results of this study showed that CO exposure was positively correlated with DED, suggesting the potential risk of regional emission sources (i.e., agricultural burning) in the incidence of DED. It was reported that SO_2_ exposure was positively correlated with decreased TBUT and OSDI scores, leading to eye sensitivity and irritation^25^. However, an association between SO_2_ and DED was not observed in our study area. One possible explanation may involve the implementation of desulfurization measures and upgrading of coal-burning power plants to achieve ultralow emissions, which significantly reduce SO_2_ emissions. In addition, the threshold for inducing DED may not be reached due to the small fluctuation range of the current SO_2_ concentration. Thus, future studies should focus on the threshold between pollutant concentration and DED in cold regions.

Our study also showed that local climatic factors (AP, AT and WS) were positively correlated with DED through epidemiological research. Versura et al. revealed that subjective discomfort symptoms were related to low corneal temperature in DED patients^38^, supporting the hypothesis that a decreased corneal temperature is involved in discomfort perception in DED patients, and this decrease is correlated with tear evaporation. The tear film lipid layer was affected dramatically at low temperatures^39^. Another in vitro study revealed that if the temperature increased from 25 to 34 °C, there was a threefold increase in the tear evaporation rate^40^. Previous studies have reported the importance of AT in DED, which is also the case in cold regions. Moreover, it is known that high altitude is usually accompanied by low air pressure. Although the relationship between AP and DED has not been reported in previous studies, our study showed a close link between DED and AP, especially the combined impacts with air pollutants (NO_2_) and another meteorological parameter (AT). This phenomenon may occur in cold regions because high-altitude exposure leads to an altered tear film, resulting in an increased tear film osmolarity (TFO) and a reduced TBUT, providing indirect proof of the effect of AP on DED^41^. Furthermore, a study conducted in the United States found that patients residing in areas of relatively high WS were less likely to develop DED^13^. It was presumed that higher WS may disperse the concentration of air pollutant particles, resulting in reduced negative effects on DED^38^. WS in this study was found to be positively associated with DED and its symptoms, and geographic diversity may explain the difference. In cold regions, the diffusion of pollutants is slow, while the dust caused by high WS may lead to the worsening of DED. Although higher wind speeds facilitate the diffusion of air pollutants, high WS above a threshold range in cold regions can induce severe dust pollution in spring, which may lead to the worsening of DED.

The year 2018 marked a turning point, following a significant change in air pollution over the past 10 years in the cold regions of Northeast China, which was preceded by frequent air pollution events, particularly affected by straw burning in autumn and winter and coal burning in winter, with the primary air pollutant of fine particulate matter. After 2018, substantial progress was made in the optimization and adjustment of agricultural open burning, industrial structure, energy structure and transport structure. Severe regional air pollution was basically eliminated, and the concentration of fine particulate matter decreased significantly close to the Chinese national air quality level 2 standard^36^. However, particulate matter pollution in some cities still occurred because of meteorological conditions and interregional transport. In contrast, O_3_ pollution is becoming more prominent, showing the characteristics of complex pollution of particulate matter and O_3_. In addition, at the highest latitude area in China, previous studies have shown that climate warming is the most significant, the intensity of abnormal events (e.g., AT, PR and WS) is stronger and the frequency is higher. Considering the association between air pollutants, meteorological conditions and DED, we suggest that the prevalence of DED will increase in the short term, but the trend will gradually decrease in the long term as a result of synergistic control of particulate matter and O_3_ pollution, as well as combined control of climate change and air pollution. However, the uncertainty of the DED trend might increase because the interaction between climate change and atmospheric aerosols is still unclear. The limitation of this study is that there are great differences in air pollution emission sources, control measures and intensity, air pollution monitoring indices and regional transportation contributions in these cities. Therefore, it is difficult to obtain comparable spatiotemporal data covering multiple cities, which impacts the generality of conclusions about the effects of air pollution on DED in cold regions. For example, although different cities have dominant roles for the same air pollutants, their emission sources are quite different. This study focused on the outdoor environment on DED and it is also important to strengthen the research to study the effect of indoor atmospheric environment on DED. What’s more, there may be lag correlation between DED and the variables of pollution and climate. Another limitation for this study is that the COVID-19 started from January, 2020, and last almost 3 years. Thus, the incidence of DED may be influenced to some extent. The readers should pay attention to this issue that this is a relatively comparative incidence data for DED that should be used carefully in the comparison with other data.

**Fig. 6:**
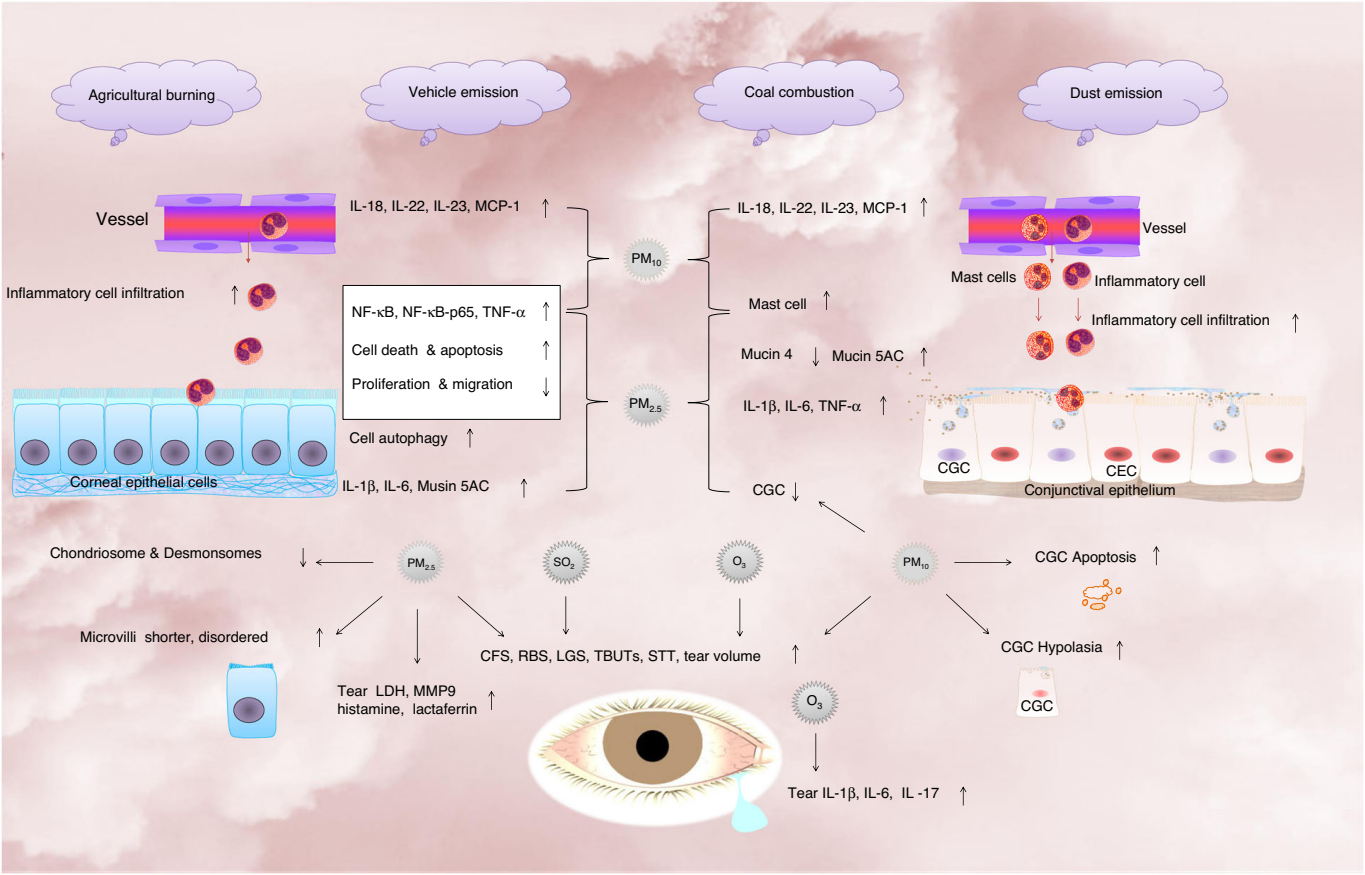
The effects and mechanism of air pollution and meteorological factors on DED. In short, PM_2.5_ decreased proliferation and migration and induced cell apoptosis and autophagy in corneal epithelial cells. PM_2.5_ significantly increased the corneal expression of NF-κB and NF-κB-p65 and the corneal or conjunctival expression of IL-1β, IL-6, TNF-α, and Musin 5AC, decreased the expression of Mucin 4 and the number of mast cells and conjunctival goblet cells (CGC) in the conjunctiva, and increased the expression of LDH, MMP9, histamine, and lactoferrin in tears. PM_10_ induced inflammatory cell infiltration and increased the expression levels of NF-κB, NF-κB-p65, and TNF-α in the cornea and IL-18, IL-22, IL-23, and MCP-1 in the cornea and conjunctiva; decreased proliferation and migration and induced cell death and apoptosis in corneal epithelial cells; reduced the numbers of chondriosomes/desmosomes in corneal epithelial cells; induced microvilli shortening and disorder; increased the number of mast cells and induced mast cell and inflammatory cell infiltration in the conjunctiva; and reduced the number of and induced hypoplasia and apoptosis in goblet cells in the conjunctiva. SO_2_ and O_3_ were positively correlated with the OSDI score and decreased TBUT. O_3_ increased IL-1β, IL-6, and IL-17 in tears and decreased conjunctival goblet cell density in a mouse model. The above results suggested that PM_2.5_, PM_10_, SO_2_, and O_3_ activated inflammation in both the cornea and conjunctiva, disrupted epithelial integrity and caused dramatic damage to the corneal epithelium and conjunctival epithelium in the development of DED

This study, for the first time, elucidates the association between DED, air pollution, and meteorological conditions in the cold region of China. It is a multidisciplinary crossover study conducted in a northeastern Chinese metropolis over 6 years. We found that DED is affected by both air pollution and climate change, with major influencing factors of atmospheric PM_10_, O_3_, and CO concentrations as well as AP, AT and WS. Worsening air quality induced by dust, agricultural open burning and O_3_ formation with a hotter windier climate is likely to trigger more cases of DED. The effects and mechanism of air pollution and meteorological factors on DED in Northeast China are elaborated (Fig. 6). Although the uncertainty of DED incidence will increase due to the interaction between climate change and atmospheric aerosols, these findings suggest the potential for a gradual reduction in DED in the future with efforts in the coordinated control of air pollution and climate change in China.

## Materials and methods

### Study area

**Fig. 7:**
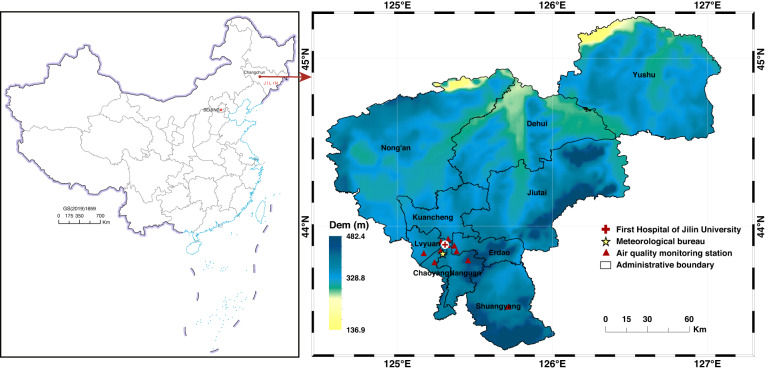
Topography, administrative division, and DED data sources in Changchun, China. Tagged represents the valid data of air pollution, meteorological factors and the DED site

The crossover study of environmental exposure and DED was conducted in the city of Changchun (Fig. 7, N43°05’-45°15’; E124°18’-127°05’), the capital of Jilin Province and the natural geographical centre of Northeast China. The region is characterized by a unique terrain (a wide heartland surrounded by three mountains), the highest latitude, and the coldest climate and suffers typical air pollution^22^. With its higher latitude, it is one of the regions with the most dramatic climate change^23^. Moreover, it is a major grain production base in China with a crop-sown area of 1.326 million hectares, resulting in intense straw open burning and large air pollutant release to the atmosphere^30,32^.

Changchun has a population of over 7.5 million, covering an administrative area of 247,446 square kilometres. The local climate is a continental monsoon with four distinct seasons, of which winter is cold and long, lasting 6 months, with an average annual temperature of 5.65 °C (−15.1 °C–23.1 °C)^30^. The metropolis belongs to a long heating cycle region with high coal burning consumption^22^, it is a typical heavy industrial city with traditional high energy consumption, high pollution, and resource-dependent enterprises and the main corn producing area with intense straw open burning, which dominate the emissions of major air pollutants.

In this study, the DED and meteorological data were collected from the First Hospital of Jilin University and meteorological monitoring sites, respectively. They are located in the Chaoyang District, the urban central region in Changchun (Fig. 7). Air quality data originated from nine state-controlled air atmospheric environment sampling sites.

### Study design and population

The present study conformed to the ethical principles of the Declaration of Helsinki. The Medical Ethics Committee of the First Hospital of Jilin University approved the study protocol (Approved No. 2022-294). First, as a retrospective study, DED data on outpatient visits from July 15, 2015, through July 30, 2021, at the Eye Center of the First Hospital of Jilin University (Changchun, Jilin Province, China), which is the largest hospital in Jilin Province, were obtained from the Information Technology (IT) Department. Outcomes were defined using the International Classification of Diseases, 10th Revision (ICD-10). The definition code for DED was H04.103, H11.104, H16.202 and H16.208. First-visit patients were included, and revisiting patients were excluded from this study. Outpatient information, including patient number, date of birth, visit date, sex, and home address, was archived. Based on the statistical analysis of residential addresses, more than 85% of the population was distributed in the city of Changchun and the surrounding areas. Females accounted for 64.60% of patients, and 35.40% were male. Patients aged between 40 and 65 years accounted for 56.17%. Accordingly, this study focused on people over 40 years of age (*n* = 5968). In addition, daily concentrations of atmospheric pollutants from national atmospheric environmental monitoring stations, daily mean or cumulative values of meteorological parameters from the National Meteorological Center, and detailed chemical components of PM_2.5_ from an automatic monitoring multipara metre station (superstation) in the city of Changchun were obtained for the same periods.

Second, to ensure accuracy and reasonableness, the original data were used for data cleaning and screening (e.g., outliers and null values) for further analysis of temporality, characteristics and relevance. The relationships among DED, air pollutants and meteorological parameters were established, and the dominant factors were screened out using multiple statistical analysis methods (e.g., correlation and regression). Furthermore, the importance and prediction of influencing factors were detected through machine learning modelling, and a crossover design was applied to explore the associations between air pollution, meteorological conditions and outpatient visits for DED. Finally, the responses of DED to air pollution and climate change, possible underlying mechanisms, source analysis and policy recommendations are proposed.

### Ambient air pollutants and meteorological factors

Six standard atmospheric pollutants (PM_10_, PM_2.5_, SO_2_, NO_2_, CO, and O_3_) were selected from nine fixed urban monitors, and the daily concentrations of these pollutants were averaged for the city. The six standard atmospheric pollutants are measured using a tapered element oscillating microbalance (PM_2.5_, PM_10_: 5030Sharp, SO_2_: 43i, NO_2_: 42i, O_3_: 49i, CO: 48i), which is a method that uses an ultraviolet fluorescence photometer, a computer digital analyser, and a nondispersive ultraviolet fluorescence photometer. For meteorological parameters, hourly mean temperature, atmospheric pressure, relative humidity and visibility were averaged, and precipitation was reported as cumulative daily values. For air quality and meteorological parameters, daily data were classified in monthly and annual scales, so as to reducing the uncertainty problem of time (daily or weekly) lag according to the analysis requirements of DED data.

The scientific research atmospheric supermonitoring station mainly performs multipollutant monitoring and comprehensively analyses the concentrations and change trends of conventional and nonconventional pollutants, secondary pollutants and precursors through various monitoring instruments and means (e.g., physical and chemical, optical, meteorological and satellite). The concentrations of anions and cations were determined by ion chromatography (ICS-1000, Dionex Inc., USA); the element concentration was obtained by inductively coupled plasma-atomic emission spectrometry (ICP-AES, IRIS, Intrepid II, Thermo Electron, USA); and quartz filters were used to determine the particulate EC and OC concentrations using a thermal-optical carbon aerosol analyser (Sunset-OCEC RT-4, Sunset Lab Inc., Tigard, OR, USA). The instrument is continuously sampled 24 h a day, and the data are recorded every 1 h.

### Statistical analysis

Data analyses and plotting were performed using R 3.4.1. DED data, indices of air quality, and meteorological parameters were matched at the same time scale for further analysis. The estimated incidence of DED in each year was based on the number of visits for DED patients and normalized by the proportion at the month level. In addition, statistical analysis (day, month, year) conformed to the severe DED indicator data, and the normalized proportion was calculated. Descriptive statistics, univariate analysis and multivariate analysis were both conducted. The significance level was set at 0.05, and all the tests were *T* tests.

Pearson’s chi-square test was applied for statistical data. In addition, grey relational analysis, which is an impacting measurement method in grey system theory that analyses uncertain relations between one main factor (i.e., DED) and all the other factors and has been adopted in public with good budget and judgement performance, was performed. A binary nonconditional logistic model was used to conduct multivariate regression analysis, and the stepwise method was adopted to select significant independent variables. The machine learning modelling method (i.e., decision tree regression) was used to simulate and predict continuous values with tree structure, and the potential correlation between environmental factors and DED was fully verified and analysed. The proportion of participation in model training was 0.7 (i.e., 70% of the data is used as training and the remaining 30% is applied to data testing).
